# Polymorphism of Melanocortin Receptor Genes—Association with Inflammatory Traits and Diseases

**DOI:** 10.3390/diseases13090305

**Published:** 2025-09-16

**Authors:** Mainak Bardhan, Ayush Anand, Amaan Javed, Maria Andrea Chilo, Nida Khan, Tulika Garg, Arihant Surana, Helen Huang, M M Samim, Vinay Suresh, Abhinav Khare, Bindu Menon, Tithishri Kundu

**Affiliations:** 1 The John P. Hussman Institute for Human Genomics, Miller School of Medicine, University of Miami, Miami, FL 33101, USA; 2 The Dr. John T. Macdonald Foundation, Department of Human Genetics, Miller School of Medicine, University of Miami, Miami, FL 33101, USA; 3 BP Koirala Institute of Health Sciences, Dharan 56700, Nepal; 4 University College of Medical Sciences, Dilshad Garden, Delhi 110095, India; 5 UAB Department of Family and Community Medicine and Cahaba Medical Care, Birmingham, AL 35205, USA; 6 Jinnah Sindh Medical University, Karachi 75510, Pakistan; 7 Government Medical College and Hospital, Chandigarh 160030, India; 8 St. Vincent Hospital, Worcester, MA 01608, USA; 9 Faculty of Medicine and Health Science, Royal College of Surgeons in Ireland, D02 YN77 Dublin, Ireland; 10 Department of Neurology, National Institute of Mental Health and Neurosciences, Bangalore 560030, India; msmondal788@gmail.com; 11 Department of Psychiatry, University of Oxford, Warneford Hospital, Warneford Ln, Headington, Oxford OX3 7JX, UK; 12 All India Institute of Medical Sciences, Gorakhpur 273008, India; 13 Department of Neurology, Apollo Specialty Hospitals, Nellore 524004, India; 14 Department of Pharmacology, Manipal Tata Medical College Jamshedpur, Manipal Academy of Higher Education, Manipal 576104, India

**Keywords:** melanocortin receptors (MCR), genetic polymorphism, inflammatory disorders, repository corticotropin injection (RCI)

## Abstract

Melanocortin receptors (MCRs) are responsible for various functions ranging from skin pigmentation, regulation of appetite, stress response and cognition, steroid synthesis, and energy balance to cellular regeneration and immunomodulation. The genetic polymorphism with tissue distribution ranging from the brain, limbic system, and adrenal cortex to neutrophils, monocytes, and macrophages is evident in MCRs. The mutations in MC1R, MC2R, MC3R, and MC4R genes are associated with risk of melanoma, familial glucocorticoid deficiency, obesity, and type 2 diabetes mellitus, respectively. Meanwhile, MC1R, MC2R, and MC5R genes are involved in the risk of major depressive disorder. Melanocortin receptors are involved in different inflammatory disorders, i.e., atopic dermatitis, autoimmune uveitis, sarcoidosis, respiratory diseases, multiple sclerosis, scleroderma, inflammatory bowel disease, amyotrophic lateral sclerosis, Alzheimer’s disease, arthritis, and reperfusion injury. Several newer therapeutic agents related to MCRs have numerous advantages over the current anti-inflammatory drugs, demonstrating therapeutic relevance. Among them, α-MSH analogs play a role in atopic dermatitis and scleroderma, and MC1R agonist Dersimelagon has shown effectiveness in systemic sclerosis. The FDA has recently approved the repository corticotropin injection (RCI) to treat sarcoidosis. The FDA has also approved various melanocortin agonists, i.e., Bremelanotide, Afamelanotide, and Setmelanotide, for the treatment of hypoactive sexual desire disorder, Erythropoietic protoporphyria, and obesity, due to pro-opiomelanocortin and leptin receptor deficiency, respectively. Therefore, this review aims to summarize the function and genetic polymorphism of melanocortin receptors, regulatory pathways involving MCRs, and the existing evidence of the prime effect of MCRs on inflammatory responses via different mechanisms and their potential therapeutic use in inflammatory diseases.

## 1. Introduction

The melanocortin system is a complex neuroendocrine and immunomodulatory network composed of multiple agonists, two antagonists, and five G protein-coupled receptors (MCRs). These endogenous agonists consist of α- Melanocyte stimulating hormone (MSH), β-MSH, γ-MSH, γ3-MSH, diacetyl-α-MSH, and adrenocorticotropic hormone (ACTH), most of which are derived from post-translational processing of pro-opiomelanocortin (POMC) [1]. In the anterior pituitary, pro-opiomelanocortin generates ACTH under the influence of prohormone convertase (PC) [1,2]. Furthermore, MSH is derived from pro-opiomelanocortin under the influence of PC2 in the skin, hair follicles, and brain. The aforementioned endogenous antagonists include agouti and agouti-related proteins (AGRPs) [3]. These antagonists interact with remaining ancillary proteins like mahogany and syndecan-3 to modulate their functions, including altered neuronal and inflammatory responses [4]. The third component of this system is the five melanocortin receptors (MCRs) numbered in the sequence of their cloning order, i.e., MC1R to MC5R [3]. Pigmentation in skin and hair follicles is modulated by MC1R [5]. MC2R synchronizes adrenal steroid formation and cell proliferation [1,6]. MC3R is required for meal intake entrainment [6]. MC4R, the most abundantly expressed melanocortin receptor in the CNS, regulates both meal consumption and energy utilization. MC4R is expressed in both neurons and astrocytes and its activation in astrocytes shows significant anti-inflammatory and neuroprotective effects in vitro [7]. MC5R regulates exocrine secretions [8,9]. These components interact and help synchronize prime physiological functions like skin pigmentation, steroidogenesis, and inflammatory reactions. These receptors are widely pronounced in the immune pathways and contribute extensively to the host’s innate defense, corroborated by their presence in the skin, respiratory tract, and gastrointestinal tract [10]. MC1R has the highest affinity for α-MSH and exists in cells mediating inflammation, i.e., monocytes, macrophages, neutrophils, mast cells, fibroblasts, and antigen-presenting cells [11,12]. Studies have also reported the involvement of MC3R and MC5R in the B lymphocyte-mediated inflammatory processes [13,14]. The activation of these cells has been shown to influence nuclear factor kappa B, which engages in the transcription signaling and modulation of genes for cytokines, chemokines, growth factors, major histocompatibility complex (MHC), anti-apoptotic factors, and inducible nitric oxide synthase [15]. Knowledge regarding the physiology and pharmacology of these melanocortin receptors can be used to treat various inflammatory disorders. A landmark study by Getting et al. showed the involvement of MC3R in gouty arthritis, where MC3R altered the efficacy of ACTH [16]. Pharmacological agents revolving around melanocortin receptor-targeted ligands offer several advantages over the current anti-inflammatory drugs. They do not alter the host defense mechanism, for example, normal host microbiota, rather they enhance the immunological process and phagocytic activity and engage various endogenous mechanisms to resolve inflammation [17,18,19]. Therefore, this review aims to summarize the genetic polymorphism of melanocortin receptors, regulatory pathways involving MCRs, and the existing evidence of the prime effect of MCRs on inflammatory responses via different mechanisms and their potential therapeutic use in inflammatory diseases.

## 2. Melanocortin System

Melanocortin peptides, melanocortin receptors, transmembrane accessory proteins, and melanocortin agonists and antagonists constitute the backbone of one of the most intricate hormonal systems in the human body, the melanocortin system [20]. The pro-opiomelanocortin gene found on chromosome 2p23 of the human genome is expressed in different tissues, i.e., the immune system cells and organs, the skin melanocytes, the gastrointestinal tract, and more predominantly in the central nervous system [21]. Pro-opiomelanocortin neurons can be distinguished as early as Embryonic Day 10 in the anterobasal area [5]. This region also includes neurons from the ventromedial nucleus of the hypothalamus, which are characterized by the presence of steroidogenic factor 1 [22]. Pro-opiomelanocortin has two groups of neurons, both derived from precursor cells located in the third ventricle’s epithelial lining [23]. A group of these neurons is present in the arcuate nucleus of the hypothalamus, whereas the other resides in the nucleus of the solitary tract [24].

**Figure 1:**
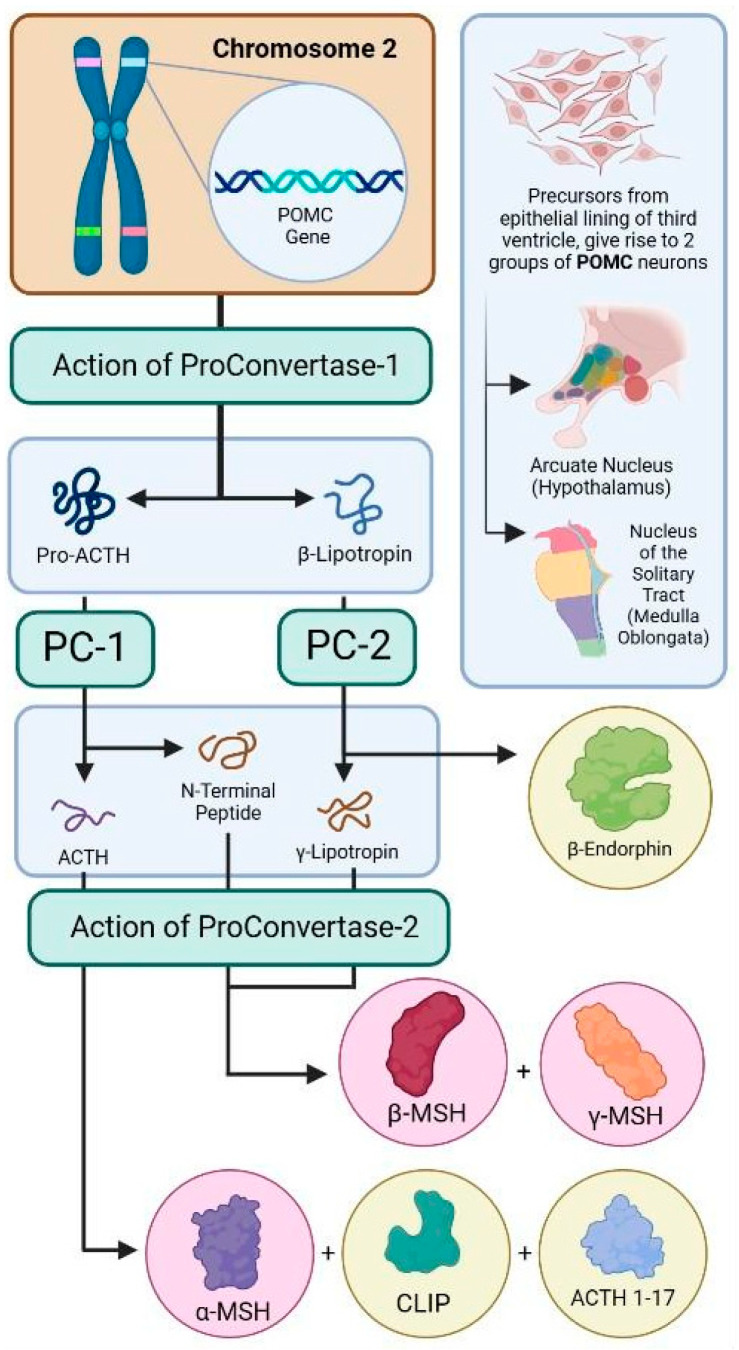
The synthesis of various melanocortin peptides from post-translational processing of the POMC gene. ‘POMC’—pro-opiomelanocortin, ‘PC-1′—Proconvertase-1, ‘PC-2′—Proconvertase 2, ‘ACTH’—Adrenocorticotropic hormone, ‘MSH’—Melanocyte stimulating hormone, ‘CLIP’—Corticotropin-like intermediate lobe peptide.

Different peptides are derived from the post-translational processing of pro-opiomelanocortin prohormone by key enzymes [24]. Pro-ACTH and β- lipotropin (β-LPH) are the products of proconvertase 1 (PC1) action on pro-opiomelanocortin. Pro-ACTH is further processed into ACTH and N terminal peptide by PC1. Furthermore, ACTH produces ACTH 1-17, corticotropin-like intermediate lobe peptide (CLIP), and α-MSH via the action of proconvertase 2 (PC2) [25]. On the other hand, β-LPH is cleaved by PC2 into γ-lipotropin (γ-LPH) and β- endorphin (β-END) [25]. Moreover, PC2 action on γ-LPH and N-terminal peptide will produce β-MSH and γ-MSH, respectively, as shown in Figure 1. Only α-, β-, γ- MSH, and ACTH constitute melanocortin peptides [26]. Each peptide exerts biological activity by displaying a specific affinity for different receptors. Five melanocortin receptors are found in various tissues (MC1R–MC5R). They are coupled to a G stimulatory protein and are all associated with cAMP production through adenyl cyclase activation. Their action ranges from skin pigmentation for MC1R, steroid synthesis for MC2R, and energy balance for MC3R and MC4R to immunomodulation for MC1R and MC5R [3].

Besides pro-opiomelanocortin derived peptides that act as agonists for melanocortin receptors, there are two known endogenous melanocortin receptor antagonists, i.e., agouti-signaling protein (ASIP) and agouti-related protein (AGRP). Their central role is blocking melanocortin peptides’ biological activity by interfering with their binding to specific receptors. ASIP and AGRP are found in the follicle melanocytes and central nervous system, respectively, and both target α-MSH by exerting their antagonizing effects on MC1R for ASIP and on MC3R and MC4R for AGRP [27,28]. Besides G-proteins and kinases, accessory proteins regulate melanocortin receptors [27]. Those two small transmembrane accessory proteins allow for the trafficking of different melanocortin receptors. MRAP1 (melanocortin receptor accessory protein 1), found in the zona fasciculata of the adrenal gland, is required for MC2R signaling, through which ACTH mediates its corticotropin action [19]. On the other hand, MRAP2 (melanocortin receptor accessory protein 2) is found in various tissues, among which the hypothalamus allows the expression of MC4R, which regulates food intake and energy homeostasis [28]. Therefore, mutations in MRAP1 and MRAP2 have been shown to result in glucocorticoid deficiency and early-onset obesity, respectively. Table 1 demonstrates the characteristic features of different types of melanocortin receptors.

## 3. Genetic Polymorphisms of Melanocortin Receptors and Their Biological Functions

Melanocortin receptors (MCRs) belong to the G protein-coupled receptors (GPCRs) family, which plays important roles in diverse physiological processes such as pigmentation, energy regulation, sexual function, and inflammation. Genetic variations in MCR genes can lead to changes in receptor function, which can affect these biological processes.

### 3.1. MC1R Polymorphism

The melanocortin 1 receptor (MC1R) gene is crucial in determining human pigmentation. Genetic polymorphisms or variations in the MC1R gene can influence receptor function and have significant implications for pigmentation phenotypes and biological responses.

#### 3.1.1. Pigmentation

MC1R polymorphisms are well-known for their association with hair, skin, and eye color. Variants in MC1R can lead to a shift in melanin production, altering the balance between eumelanin (dark pigment) and pheomelanin (red/yellow pigment). Red hair color, fair skin, and increased sensitivity to ultraviolet (UV) radiation are common characteristics associated with certain MC1R polymorphisms [29,30].

#### 3.1.2. UV Radiation Response

MC1R plays a crucial role in regulating the response to UV radiation. Activation of MC1R by its ligand α-MSH promotes eumelanin synthesis and protects against UV-induced DNA damage. However, certain MC1R polymorphisms reduce MC1R signaling, leading to decreased eumelanin production and increased susceptibility to sunburn and skin cancer [29,31].

#### 3.1.3. Inflammation and Immune Modulation

MC1R has been implicated in immune modulation and inflammation regulation. Activation of MC1R can influence the production of cytokines and immune mediators, thereby modulating immune responses and inflammation. MC1R polymorphisms have been associated with altered immune response profiles and may contribute to variations in susceptibility to autoimmune diseases and skin disorders [29].

#### 3.1.4. Risk of Melanoma

Certain MC1R polymorphisms are considered as genetic risk factors for melanoma, a type of skin cancer. Variants associated with red hair color and fair skin, such as R151C, R160W, and D294H, have increased susceptibility to melanoma [29,32]. These variants are associated with reduced MC1R function and altered melanin synthesis, contributing to an increased risk of developing melanoma.

### 3.2. MC2R Polymorphism

The MC2R gene encodes the ACTH receptor, which is essential for adrenal gland function and cortisol synthesis, a hormone involved in stress response and metabolism. Genetic polymorphisms or variations in the MC2R gene can result in altered receptor function, significantly affecting adrenal function and cortisol synthesis.

#### 3.2.1. Familial Glucocorticoid Deficiency (FGD)

Mutations in the MC2R gene can cause FGD, a rare autosomal recessive disorder characterized by impaired cortisol synthesis and adrenocortical insufficiency. FGD is typically associated with homozygous or compound heterozygous mutations in MC2R, leading to dysfunctional MC2R receptors with reduced binding affinity for ACTH [33]. As a result, cortisol production is impaired, leading to symptoms such as fatigue, hypoglycemia, and electrolyte imbalances [34].

#### 3.2.2. Impaired Cortisol Response

MC2R polymorphisms that result in altered receptor function or reduced ACTH binding affinity can impair cortisol response to stress or ACTH stimulation [33]. These variations can affect the signaling and downstream activation of the hypothalamic–pituitary–adrenal (HPA) axis, resulting in suboptimal cortisol production in response to physiological demands.

#### 3.2.3. Steroidogenesis

MC2R is crucial in regulating steroidogenesis, particularly cortisol synthesis in the adrenal cortex. The activation of MC2R by ACTH stimulates various enzymatic reactions that are involved in synthesizing cortisol from cholesterol [35]. Genetic polymorphisms in MC2R can disrupt this process and impair cortisol production, as observed in FGD.

### 3.3. MC3R Polymorphism

The MC3R gene is predominantly present in the hypothalamus and is involved in energy homeostasis, regulation of food intake, and metabolic processes. Genetic polymorphisms or variations in the MC3R gene can influence receptor function and have significant implications for body weight regulation and obesity.

#### 3.3.1. Body Weight Regulation

MC3R regulates meal consumption, utilization of energy, and body weight. Activation of MC3R signaling in the hypothalamus leads to reduced meal consumption and increased energy expenditure [36]. Genetic polymorphisms in MC3R can impact receptor function and alter these regulatory mechanisms, potentially leading to variations in body weight and risk of obesity.

#### 3.3.2. Risk of Obesity

Several studies have investigated the association between MC3R genetic polymorphisms and obesity. Certain variants, i.e., V81I and I172V, are related to increased body mass index (BMI) and a higher chance of obesity [37]. These polymorphisms can affect MC3R signaling and disrupt the normal regulation of consumption of meal and energy balance, contributing to increased susceptibility to weight gain and obesity.

#### 3.3.3. Appetite Control

MC3R is involved in the central regulation of appetite and satiety. Genetic variations in MC3R can influence sensitivity to satiety signals and impact the regulation of food intake [38]. Altered MC3R function resulting from genetic polymorphisms can disrupt appetite control mechanisms, potentially leading to overeating and weight gain.

### 3.4. MC4R Polymorphism

The MC4R gene is pivotal in regulating energy homeostasis and appetite. Genetic polymorphisms or variations in the MC4R gene can influence receptor function and have significant implications for body weight regulation, satiety, and the risk of obesity.

#### 3.4.1. Regulation of Appetite

MC4R is primarily expressed in regions of the brain, i.e., the hypothalamus, which is associated with regulation of appetite and energy balance. Activation of MC4R signaling promotes satiety and reduces food intake [39]. Genetic polymorphisms in MC4R can affect receptor function and disrupt the normal regulation of appetite, leading to dysregulated eating behaviors and an increased risk of overeating and obesity.

#### 3.4.2. Risk of Obesity

MC4R mutations and genetic variants have been identified as the most common monogenic causes of severe early-onset obesity. Loss-of-function mutations or variants that impair MC4R signaling can disrupt the normal satiety signaling pathways, leading to hyperphagia and obesity [40,41]. In contrast, certain rare gain-of-function mutations in MC4R have been associated with reduced appetite and protection against obesity [42].

#### 3.4.3. Metabolic Effects

MC4R signaling also influences metabolic processes beyond appetite regulation. Activation of MC4R promotes energy expenditure and thermogenesis [38]. Genetic variations in MC4R may impact these metabolic effects, contributing to energy expenditure and variations in metabolic rate.

### 3.5. MC5R Polymorphism

MC5R serves multiple vital functions in both central and peripheral tissues. In the brain, MC5R plays a role in stress response, cognitive function, and fetal brain development. In the perifornical lateral hypothalamus, MC5R may control physical activity in lean rats. In peripheral tissues, MC5R involves various processes such as exocrine and endocrine gland secretion, defense behavior, thermoregulation, inflammation, and immune response [43]. MC5R regulates energy metabolism in different species, including humans, mice, chickens, and sea bass, primarily by affecting lipolysis of adipose tissue and re-esterification, fatty acid oxidation, and glucose uptake in the liver, adipose tissue, and skeletal muscle [44].

MC5R plays a role in sebogenesis by regulating thermal and sebaceous lipid production with water repulsion. It is associated with sebum production and sebocyte differentiation. In mice with MC5R knockout, the production of sebaceous lipids is downregulated [44]. Ocular immunity is achieved by Alpha-MSH, which, through MC5R, mediates CD4+ regulatory T cells induction, which suppresses autoimmune disease and also protects the retina from inflammatory damage [45]. It regulates the immune response by inhibiting proinflammatory signals in macrophages [8].

MC5R is a modulator of the responses stimulated by glucose in ARPE-19 cells and can modulate the response of retinal pigment epithelium to diabetes in vivo [46].

The recent literature shows that three genes (MC1R, MC2R, and MC5R) increase the risk of major depressive disorder (MDD), and one gene (MC4R) is associated with an increased risk of type 2 diabetes, but its association needs to be researched further [47].

Table 1 summarizes the polymorphisms of each melanocortin receptor subtype, their primary biological roles, and associated disease risks. This complements the individual discussions of MC1R–MC5R and underscores both their distinct and overlapping roles in inflammatory and metabolic pathologies.

## 4. Intracellular Signaling

### 4.1. G Protein-Coupled Receptor (GPCR) Mediated Signaling

Melanocortin mainly acts via melanocortin receptor (MCR) which is a G protein-coupled receptor (GPCR). GPCRs have three subunits, i.e., α, β, and γ. GPCRs are divided into three types based on their alpha subunit, i.e., Gs, Gi, and Gq. All melanocortin receptors are Gs subtype and increase the level of cAMP and act via protein kinase A pathway (PKA) [48]. MC3R, MC4R, and MC5R receptors bind to Gi receptors, whereas MC4R is also associated with the Gq subtype.

#### 4.1.1. Melanocortin 1 Receptor (MC1R)

α MSH binds to the Gs subtype of GPCR (MC1R receptor) and enhances the level of cAMP, acting via protein kinase A pathway and leading to an increased Ca^2+^ level, which acts as a second messenger [48,49]. Increased cAMP leads to activation of Rap1 and Ras, leading to B-Raf signaling, ERK1/2 phosphorylation, and melanin synthesis. In human melanoma cell line HBL, binding of the ligand to MC1R causes the transactivation of c-KIT leading to the ERK1/ERK2 mitogen-activated protein kinase (MAPK) pathway, resulting in decreased melanin formation, which is independent of cAMP [50]. Therefore, in MC1R mutant human melanoma cells, cAMP-dependent activation is disrupted, but the ERK1/ERK2 MAPK pathway functions, leading to a protective role in skin cancer. In the human retinal pigment epithelium cell line, binding of α MSH to MC1R can cause the activation of Akt–mTOR signaling and diminished oxidative stress and risk of age-related macular degeneration (AMD) [51]. The binding of ACTH to MC1R is known to cause p38 pathway activation in human keratinocyte cells [3].

#### 4.1.2. Melanocortin 2 Receptor (MC2R)

ACTH binds to MC2R and is responsible for steroid hormone synthesis from the adrenal gland. The MC2R receptor is exceptional, as it binds to only ACTH, whereas the other MCRs have affinity for both ACTH and MSH. Like the MC1R receptor, when the ligand ACTH binds to the Gs subtype of GPCR (MC2R receptor), increased cAMP and activation of the protein kinase A pathway occur. Similarly, like the MC1R receptor, the binding of ACTH to the MC2R receptor is known to cause p38 pathway activation in human keratinocyte cells [3]. MC2R also acts via protein kinase C (PKC) and ERK1/ERK2 MAPK pathway. Whether ERK1/ERK2 activation is associated with protein kinase A pathway is controversial. Some studies show that ERK1/ERK2 activation is protein kinase A dependent whereas other literatures have revealed that the activation of ERK1/ERK2 is independent [52]. After ACTH binds to its receptor, slow and sustained enhancement of Ca^2+^ level is responsible for its proper function [53]. Desensitization and receptor internalization are related to the short-term regulation of MC2R. While protein kinase A and PKC are related to the desensitization of MC2R, only protein kinase A pathway is needed for internalization [54]. ACTH-mediated activation of both the kinases, i.e., protein kinase A and MAPK, requires the transactivation of steroidogenic factor 1 (SF1) [55].

#### 4.1.3. Melanocortin 3 Receptor (MC3R)

MC3R is associated with cell proliferation, regeneration of neuronal cells, and myocardial reperfusion [56]. Similarly to the other MCRs, MC3R is also of the Gs subtype. When ligands bind to MC3R, it leads to an increased cAMP level and activation of the protein kinase A pathway, leading to an enhanced level of Ca^2+^. This receptor can also attach to Gi and acts via PI3 activation and the ERK1/2 pathway [57].

#### 4.1.4. Melanocortin 4 Receptor (MC4R)

MC4R is related to regulation of meal consumption and appetite, utilization of energy, thermogenesis, and development of type 2 diabetes mellitus. MC4R is unique as it attaches to Gs, Gi, and Gq. Like other MCRs, when ligands bind to MC4R, it leads to an increased cAMP level and activation of the protein kinase A pathway, leading to an enhanced level of Ca^2+^ [58]. MC4R also binds to Gq and acts via PLCβ and IP3, leading to the activation of PKC and an increased Ca^2+^ level. MC4R also attaches to Gi and acts via ERK1/2. In GT1 cell line, activation of ERK1/2 is associated with anti-apoptosis [59]. Activation of PI3K, PKC, and protein kinase A can be related to ERK1/2 activation. In HEK293 cell culture, melanocortin agonists bind to MC4R and cause the subsequent reduction and enhancement of phosphorylation of IRS-1ser307 and AKT, respectively. This mechanism is responsible for c-jun-N-terminal kinase (JNK)-mediated regulation of the insulin signaling pathway [60].

#### 4.1.5. Melanocortin 5 Receptor (MC5R)

MC5R regulates energy metabolism primarily by affecting lipolysis in adipose tissue and re-esterification, fatty acid oxidation, and glucose uptake in the liver, adipose tissue, and skeletal muscle. Like other MCRs, it acts via both Gs and Gi. When the ligand α-MSH binds to MC5R, it activates both the Gs-cAMP-protein kinase A pathway and the Gi-MAPK-ERK1/2 pathway. α-MSH-mediated ERK1/2 activation occurs via PI3 [61]. This pathway leads to lipolysis and re-esterification, leading to decreased adipose tissue. α-MSH mediated activation of ERK1/2 can cause phosphorylation of p90RSK and stress-activated protein kinase 1 (MSK1). α-MSH acts via the cAMP-protein kinase A pathway, leading to activation of CREB [62]. Activation of MC5R via several ligands, i.e., α-MSH, ACTH, etc., is associated with a ryanodine-receptor-mediated increase in the Ca^2+^ level [63].

## 5. G Protein Independent Signaling

As a rule, GPCR acts via G protein-coupled pathways. But recent studies show that some GPCRs, i.e., β2 adrenergic receptor and melanocortin receptor, act via G protein-independent pathways, i.e., JAK/STAT, PDZ domain-containing proteins, etc. [64]. Binding of α -MSH to MC1R results in activation of the ERK1/ERK2 mitogen activated protein kinase (MAPK) pathway. This activation is mediated by the transactivation of cKIT belonging to receptor tyrosine kinases. The MC1R variant related to an increased risk of skin cancer is mediated by decreased levels of cAMP. The ERK1/ERK2 MAPK pathway is distinctive as it involves transactivation of cKIT instead of involving cAMP [50]. In the Ba/F3 cell line, the binding of α-MSH to MC5R can lead to activation of the JAK/STAT pathway [65]. In 3T3-L1 adipocytes, an enhanced level of IL6 is associated with the binding of α-MSH and ACTH with MC5R and MC2R, respectively [66].

## 6. β-Arrestin Dependent Signaling

β-arrestin is associated with the desensitization and internalization of GPCRs. After the ligand binds to GPCR, phosphorylation via PKA or PKC occurs, followed by binding of β-arrestin and desensitization of the receptor [54]. Clathrin-coated-pits (CCPs) play an important role in receptor desensitization. β-arrestins recruit the clathrin molecules to form CCPs. Desensitization of GPCR is usually followed by receptor internalization and downregulation. Other than their role in GPCR desensitization and internalization, β-arrestin is also responsible for the formation of scaffold and signal transduction [62]. β-arrestin 2 is responsible for the desensitization and internalization of MC1R. Binding of the ligand α-MSH to MC5R can lead to biphasic activation of the ERK1/2 pathway. This pathway is characterized by initial transient Gi-mediated activation followed by β-arrestin-mediated late sustained activation [61].

## 7. The Melanocortin Receptor Activation Effect on Anti-Inflammatory Cells and Responses

The melanocortin receptor activation effect on anti-inflammatory cells has been an ongoing study for more than a decade on mice, rats, rabbits, and human organisms. Various studies demonstrated that ACTH acts through the activation of melanocortin receptors found in the brain and peripheral immune cells to decrease inflammation. The receptors also possess a specific function in regulating melanocortin amino acids. A study in vitro of α-MSH was shown to inhibit nuclear factor-kB (NF-kB) activity [3]. NF-kB is a transcriptional regulatory protein that modulates the manufacturing of inflammatory cytokines, chemokines, cell cycle regulators, and adhesion molecules. It is also an activator of the immune system cells [67].

Manna and Aggarwal were the first scientists to discover that MSH activation suppresses TNF-mediated NF-kB in an Electrophoretic Mobility shift assay. MSH activation also represses NF-kB activity mediated via lipopolysaccharides and ceramides in the human epithelium, glioma, lymphoid, and monocyte cells. According to further studies, it was discovered that the α-MSH effect was mediated through the activation of CAMP, which inhibits adenylyl cyclase, suggesting that this is one of the mechanisms of the anti-inflammatory effect [68]. A study conducted in human monocytic leukemia cell lines and macrophage lines showed the inhibition of inflammatory reactions in the presence of α-MSH and its action on human melanocortin 1 receptor (hMC1R), hMC3R, and hMC5R [69]. In a rodent study, the receptor MC5R was isolated in the spleen thymus, lacrimal gland, and adrenal tissue, and took part in the effects of ACTH on immune cells [70].

Melanocortin receptors have also been studied in the brain. In a gerbil and rat model, MC4R provided neuroprotection in cerebral ischemia, suggesting a role in the inflammatory cascade [71]. In the human glial cells, there are receptor subtypes 1, 3, 4, and 5, yet the only receptor involved with inflammation is MC4R in a rat astrocyte [8]. Astrocytes are neuron cells involved in the inflammation process of Multiple Sclerosis and Alzheimer’s disease. The cascade of inflammatory reactions influences α-MSH and MC4R. Binding of α-MSH with MC4R decreases the intensity of neurodegenerative diseases by stopping the lipopolysaccharide and interferon gamma mediated cell death in astrocytes [72].

MC4R modulation presents a promising avenue for therapeutic intervention in various neurodegenerative conditions. Melanocortin peptides have demonstrated the ability to protect neurons in both acute and chronic experimental neurodegenerative conditions. This suggests that the modulation of MC4R could potentially protect against neuronal damage in conditions such as ischemic stroke, traumatic brain injury, spinal cord injury, and Alzheimer’s disease. Melanocortin activates crucial signaling pathways and thus promotes neurogenesis, which could be beneficial for conditions where neuronal loss is a prominent feature. Experimental models have consistently shown that melanocortin-induced modulation leads to permanent recovery in synapse activity and neurological performance, i.e., learning and memory, sensory-motor orientation, etc. Other than its neuroprotective and neurogenetic action, melanocortin also has a role in counterbalancing systemic responses generated due to brain injury, indicating a broader protective role beyond direct neuronal effects. All these effects are primarily mediated by brain MC4R and the vagus nerve. The observed effects in animal models suggest that MC4R agonists could be valuable as neuroprotective and neurodegenerative agents in various neurodegenerative diseases, addressing a significant unmet medical need, such as in multiple sclerosis (MS). Setmelanotide is one MC4R agonist, and has been found to markedly induce interleukin −6 and −11 production in astrocytes, possibly through enhanced cAMP response element-binding protein (CREB) phosphorylation. This suggests a potential mechanism for its anti-inflammatory effects [7]. Mcr4 agonists have a valuable role in neurodegenerative diseases. Preclinical evidence suggests that MC4R affects peripheral and central functions; pain, appetite, memory, and fever [73]. Activation of MC4R is also associated with neuroinflammation, leading to reduced brain edema in mice and improved neurobehavioral activities. It has potential in the management of intracerebral hemorrhage according to Chen et al. [74]. Its ability to enhance neutrophil activity and antipyretic effects is effective in the treatment of stroke, subarachnoid hemorrhage, and Alzheimer’s disease, and it enhances neurogenesis [73]. The phase 3 trial supported the efficacy of mcr4 agonists in treatment of obesity in Bardet–Biedl syndrome [75].

While these findings strongly support a mechanistic role for MC4R in neuroinflammation, it is important to emphasize that the evidence remains preclinical. No Phase II or III clinical trials have yet evaluated MC4R agonists in multiple sclerosis, Alzheimer’s disease, or related conditions. The only MC4R-targeted therapy with regulatory approval to date is setmelanotide, which is indicated for rare monogenic obesity syndromes and not for neurological diseases. Although setmelanotide has been shown to induce IL-6 and IL-11 production in astrocytes in vitro, translating these observations into clinical benefits remains untested [7].

Therefore, the current body of evidence should be interpreted as hypothesis-generating rather than clinically validated. MC4R agonists represent a promising therapeutic avenue, but their efficacy in neurodegenerative disease remains to be demonstrated through well-designed clinical trials.

## 8. Association of Melanocortin Receptors in Therapeutic Management of Inflammatory Disorders

### 8.1. Role of Melanocortin Receptors in Cutaneous Disorders

Chen et al. assessed the effect of MC1R on inflammation in atopic dermatitis mice models and found that MC1R can reduce inflammation and be targeted as a therapeutic modality in dermatological diseases such as dermatitis [76]. Andoh et al. reported a significantly increased level of α-MSH in epidermal keratinocytes of atopic dermatitis patients [77]. The skin of mice mainly consisted of MC1R, MC2R, and MC5R mRNAs [77]. In these mice, α-MSH induced scratching [77]. A study by Hiramoto et al. shed light on how exercise and α-MSH levels can affect the exacerbation of dermatitis. They reported decreased α-MSH levels and MC1R expression after mild exercise but increased levels after intense exercise. This suggests the involvement of α-MSH in the exacerbation of dermatitis [78].

Though the exact mechanism could not be delineated, α-MSH production-related enzymes and receptors could help develop drugs with antipruritic action [79]. A study by Luo et al. supported the finding of α-MSH action via the MC1R. They found reduced expression of MC1R in dermal fibroblasts in keloid scars [79]. Hence, restoring the MC1R receptors can be explored as a therapeutic approach to decreasing scar formation.

### 8.2. Role of Melanocortin Receptors in Cardiovascular Disorders

Hart Sailors et al. pointed toward the role of melanocortin receptor genes in atherosclerosis [80]. Rinne et al. investigated the role of the melanocortin system on atherosclerotic plaque and reported that the melanocortin system could decrease plaque formation and limit vascular endothelial dysfunction in atherosclerosis [81]. Lede et al. reported hyperphagia leading to obesity and increased risk of atherosclerosis in MC4R-deficient mice [82]. Another study delineated the role of MC1R in cholesterol transport in macrophages [83]. They found that MC1R activation can decrease macrophage foam cell formation. Also, MC1R deficiency can promote atherosclerosis in mice [84]. A recent study revealed that melanocortin activation could improve glucose regulation and diminish vascular inflammation and plaque formation [85]. Though MC3R activation with γ-MSH significantly reduced inflammation, its role in reducing plaque is still insignificant [86].

### 8.3. Role of Melanocortin Receptors in Ophthalmic Disorders

The melanocortin system has a significant role in mediating autoimmune uveitis. Lee et al. showed that subconjunctival injection of ACTH1-17 plasmid increased α-MSH in the aqueous humor of mice and attenuated inflammation [87]. Edling et al. suggested the anti-inflammatory action of α-MSH analog in experimental autoimmune uveitis [88]. Also, the regulatory immunity in uveitis can be mediated via MC5R and adenosine 2 A receptor (A2Ar) [89,90]. Obesity can potentially lead to the worsening of autoimmune uveitis through MC5R [91]. Recent studies have suggested that MC1R and MC5R can be therapeutic targets to suppress autoimmune uveitis [8,92]. The melanocortin system is also involved in maintaining the structural integrity of the retina [93].

### 8.4. Role of Melanocortin Receptors in Respiratory Disorders

Melanocortin receptors can be used as therapeutic targets in various respiratory conditions. Getting et al. observed that wild-type alveolar macrophages are expressed in both MC1R and MC3R and thus noted the potential role of melanocortin receptors in lung inflammation. In addition, they reported that melanocortin peptides could inhibit leucocyte accumulation via various pathways in allergic and non-allergic models of inflammation [94]. Overall, they showed that selective MC3R agonists could be a therapeutic modality to reduce lung inflammation [8]. Another study by Raap et al. aimed at understanding the impact of melanocortin receptors on inflammatory respiratory diseases [95]. They found an increase in α-MSH production in the lungs during allergic airway inflammation. This is the first study to report α-MSH in the lungs. To further understand the interaction of α-MSH, they treated the mice with α-MSH peptide and found significantly decreased levels of allergen-specific IgE, IgG1, and IgG2a. Also, they reported that α-MSH anti-inflammatory effects are mediated via IL-10. Overall, α-MSH can be employed as a therapeutic target in allergic airway diseases. The use of α-MSH is not only limited to lung inflammation. A study by Colombo et al. assessed the effect of α-MSH in acute lung injury in rats [96]. Similarly to the study by Raap et al., they reported α-MSH production in the lungs. Treatment with α-MSH analog significantly reduced edema, alveolar wall thickness, and inflammatory cells. Overall, there were significant anti-inflammatory effects at clinical and molecular levels. Xu et al. also reported similar results on using STY39, α-MSH analog [97]. Deng et al. studied the effect of α-MSH on lung injury after renal ischemia in a mice model [98]. Similarly to the study by Colombo et al., α-MSH led to decreased edema four and eight hours after renal ischemia. Also, α-MSH-receiving animals demonstrated significantly reduced serum creatinine levels and necrosis. Additionally, α-MSH-receiving animals showed decreased leucocyte accumulation, and reduced TNF-α and ICAM1. Overall, these findings suggest a significant role of α-MSH in renal and lung injury and the possibility of using it as a therapeutic target. Kristensen et al. conducted a study on pigs to further understand the role of α-MSH in systemic inflammatory conditions [99]. They first induced systemic inflammatory response syndrome (SIRS) using lipopolysaccharide in pigs and recorded hemodynamic responses to delineate the effects of α-MSH analog. The pigs treated with α-MSH analog had significantly delayed peak pulmonary pressure, sustained increased pulmonary vascular resistance, and prevented cardiac fractional shortening. These parameters suggest a beneficial role of α-MSH in a systemic inflammatory response. Despite various studies being conducted to understand the pathways involved in the anti-inflammatory effect of α-MSH, there are discrepancies among these studies [100]. Hence, further studies are required to delineate these pathways.

### 8.5. Role of Melanocortin Receptors in Sarcoidosis

Sarcoidosis is a multisystem inflammatory disorder affecting almost all organs. The FDA has approved only prednisolone and repository corticotropin injection (RCI) for managing sarcoidosis. The rest of the intervention approaches are based on low-quality evidence [101]. RCI is an agonist of all five melanocortin receptors [102]. It binds to MCRs and reduces inflammatory responses. The first use of RCI was reported by Miller et al., in which there was marked clinical improvement of sarcoidosis, including uveitis [103]. A retrospective study reported an objective improvement in approximately one third of advanced sarcoidosis patients on three months of using Acthar gel, an ACTH-based therapy [104]. Another prospective study found significant improvement in pulmonary status, chest X-ray, and steroid-sparing effect due to RCI use in advanced sarcoidosis patients [105]. Chopra et al. also found a significant decrease in corticosteroid level after RCI use. Also, there was a symptomatic improvement, improved lung function, and reduced inflammation [106]. We will obtain high-quality evidence once the PULSAR trial is completed [107].

### 8.6. Role of Melanocortin Receptors in Fibrotic and Sclerotic Disorders and Associated Neuroprotection

Besides sarcoidosis, various fibrotic and sclerotic conditions have been linked to melanocortin receptor pathways. Melanocortin receptors can have a potential role in the etiology of multiple sclerosis (MS) [108,109]. In addition, MC1R single nucleotide polymorphism can also affect MS outcomes [110]. Melanocortin receptors can attenuate the synthesis of extracellular matrix in scleroderma [111]. Patridge et al. reported an increased risk of MS and MS-linked disability in MS patients with MC1R His294-encoding alleles [112]. Kokot et al. investigated the effects of α-MSH using a bleomycin (BLM) mice model with scleroderma [113]. They reported decreased expression of type-I and type-III collagen in human dermal fibroblasts on treatment with α-MSH. Also, α-MSH decreased BLM-induced oxidative stress. Also, the expression of MC1R receptors in the skin suggested that this α-MSH action is MC1R-mediated. Overall, there was decreased fibrosis and collagen. A study by Kondo et al. reported that Dersimelagon, an MC1R agonist, significantly decreased inflammation, vascular dysfunction, and fibrosis in systemic sclerosis [114]. In addition, ACTH-based therapies can also be used as potential therapies for systemic sclerosis [115,116]. Systemic sclerosis can lead to inflammatory changes in the nervous system. Benjamins et al. reported a dose-dependent protective effect of ACTH in glial cells, most probably via melanocortin receptors [117]. Another study found that ACTH 1-39 increases the number of oligodendroglia precursors and maturation and reduces the death of these precursors [118]. Other studies also reported a similar neuroprotective effect of ACTH 1-39, which may be mediated via astroglia [119,120]. A recent study showed that melanocortin receptors present in oligodendroglia and oligodendroglial precursors activated ACTH-mediated neuroprotective pathways [121]. Kamermans et al. evaluated the potential role of MC4R agonists and reported significant reduction in inflammation related neurodegeneration in multiple sclerosis [7]. Mykicki et al. also reported the role of MC1R in neuroinflammatory conditions such as systemic sclerosis [122].

### 8.7. Role of Melanocortin Receptors in Gastrointestinal Disorders

α-MSH also has a role in inflammatory bowel disease [123]. Kannengiesser et al. reported prompt recovery and return of body weight to normal in the DSS colitis model after treatment with melanocortin-derived peptide [124]. Also, these mice had reduced inflammatory changes upon histological examination. Bettenworth et al. also pointed toward the promising result of melanocortin-derived peptides in inflammatory bowel disease in reducing inflammation and maintaining intestinal barrier function [125]. Nesfation-1, which acts via melanocortin system pathways, leads to significant anti-inflammatory action in colitis in rat models [126]. Dodd et al. investigated the role of a new oral formulation of a selective MC1R agonist and found promising results in ulcerative colitis [127]. Rats managed with oral formulation reported lower macroscopic colon damage and fecal occult blood and improved colon weight and stool consistency. These findings point toward the possible role of melanocortin agonists in inflammatory bowel disease.

### 8.8. Role of Melanocortin Receptors in Neurodegenerative Disorders

Other neurodegenerative disorders, such as Amyotrophic lateral sclerosis and Alzheimer’s disease, are also associated with a defect in the melanocortin system [128]. Recent studies have identified potential therapeutic targets in Alzheimer’s disease using melanocortin system pathways. Giuliani et al. studied the role of MC4R agonists in the transgenic mice model treated with NDP-α-MSH. They reported reduced phosphorylation in cerebral cortex and hippocampus and decreased levels of Alzheimer’s disease-related biomarkers after treatment with NDP-α-MSH [129]. There was also a significant decrease in the loss of neurons and improved cognitive function in these mice. This study also pointed towards the essential role of MC4R agonists in neurogenesis. MC1R gene variants are also associated with the risk of Alzheimer’s disease [130]. Lau et al. reported that MCR activation leads to the suppressed activity of the A1 subtype of reactive astrocytes. These astrocytes mediate neurotoxicity, and suppression of activity promotes neuroprotection [131]. Also, the activation of melanocortin receptors leads to homeostasis. NDP-α-MSH, a melanocortin analog, also leads to significant cognitive improvement in the advanced stages of the mice model [132]. Overall, melanocortin receptors can be employed as therapeutic targets for Alzheimer’s disease.

### 8.9. Role of Melanocortin Receptors in Hepatic Disorders

Melanocortin receptors can be explored as a therapeutic target in liver disease, such as non-alcoholic steatohepatitis (NASH). A study by Itoh et al. reported the development of NASH in melanocortin 4 receptor-deficient mice (MC4R-KO) when kept on a high-fat diet. In addition, MC4R-KO mice developed hepatocellular carcinoma when kept on a high-fat diet for up to one year [133]. Also, these mice showed increased inflammation in adipose tissue, which may contribute to liver fibrosis. Another study also looked at the role of aggregates of macrophages called hepatic crown-like structures (hCLS) in the development of NASH [134]. Additionally, the number of aggregates was directly associated with ballooning degeneration. Lee et al. conducted a study to assess the role of α-MSH in liver fibrosis. They found that α-MSH gene therapy can reverse carbon tetrachloride-induced liver fibrosis in mice [135].

### 8.10. Role of Melanocortin Receptors in Bone Diseases

Most of the subtypes of melanocortin receptors (MC1R–MC4R) are present in bone osteoblast, osteoclast cells, cartilage, and synovium [136]. POMC-derived hormones regulate bone remodeling via glucocorticoid-mediated indirect pathways and by direct effect [137]. Melanocortin receptors have a significant role in bone diseases, specifically arthritis. α-MSH targets chondrocytes and mediates inflammation and degenerative changes [138]. Other studies have highlighted the role of α-MSH in decreasing bone volume [139,140]. α-MSH has recently evolved as a unique anti-inflammatory agent for rheumatoid arthritis. But α-MSH can be a potential hindrance to fracture-healing due to its anti-inflammatory effect. Graue et al. eradicated this concern and showed that α-MSH does not have any detrimental effect on fracture repair [141]. Getting et al. reported that macrophages present in the knee joints of rat models expressed MC3R, and MC3R antagonists blocked the ACTH-mediated anti-inflammatory action. This suggested a possible role of melanocortin receptors in experimental gouty arthritis [16]. Other studies also showed similar action via MC3R pathways in experimental arthritis [142,143]. A recent study by Lorenz et al. highlighted the protective role of MC1R against cartilage destruction and subchondral bone sclerosis [144]. Activation of MC1R and MC3R via agonists can be used to counter inflammatory and degenerative changes in rheumatoid and osteoarthritis [145]. Other than several types of arthritis, melanocortin agonists can play a role in preventing alveolar bone resorption in severe types of periodontitis caused by *Aggregatibacter actinomycetemcomitans* [146].

### 8.11. Role of Melanocortin Receptors in Reperfusion Injuries

The melanocortin system plays a significant role in reperfusion injuries. Earlier studies have indicated that the melanocortin system mediates anti-inflammatory response via central and peripheral action [122,147,148]. Also, melanocortin receptors are present in the brain, facilitating the action of α-MSH. Bazzani et al. assessed the effect of melanocortin peptides on myocardial ischemia in rats. They found a significant reduction in postischemic perfusion-induced arrhythmia and mortality due to arrhythmia in rats treated with ACTH-1-24 [149]. There was a significant reduction in myocardial tissue damage on permanent coronary artery occlusion in rats treated with NDP-MSH. Also, NDP-MSH can have a role in ischemic preconditioning [150]. Various studies were conducted to delineate the receptors involved in the protective effect of melanocortin in myocardial ischemia/reperfusion and found that MC3R was the main receptor mediating this effect [151,152,153]. Additionally, melanocortin peptide HP228 also shows a protective effect in myocardial reperfusion [154]. Studies have suggested the role of the efferent cholinergic pathway in the protective effect of the melanocortin system in myocardial reperfusion [155]. Also, studies have pointed towards the role of JAK/STAT signaling and ERK signaling pathways in the protective effect of melanocortin against myocardial reperfusion injury [156,157,158]. Melanocortin also has a role in renal reperfusion injury. Lee et al. showed that α-MSH, through its action via MC1R and MC3R, can significantly decrease the effects of renal reperfusion injury in rats [159]. A recent study also showed that NDP-MSH treatment could improve lung perfusion [160]. Leoni et al. provided the evidence of the role of MC3R agonists in mesenteric microcirculation in mice [161]. Liu et al. showed the role of MC4R agonists in intestinal injuries [162]. Though earlier studies pointed towards the role of melanocortin in the protective effect of reperfusion injury, Regan et al. reported no significant neuroprotective effects in a rat stroke model using selective MC4R activation [163]. Recent studies have reported that synthetic melanocortin derivatives have a significant neuroprotective effect by decreasing inflammation and promoting the expression of neurotransmitter-related genes [164,165]. Holloway et al. showed the neuroprotective effects of MC1R and MC3R in cerebral ischemia–reperfusion [166]. Another study examined the role of melanocortin in testicular ischemia [167]. They found that selective MC4R agonists improved spermatogenesis, decreased organ damage, and inhibited apoptosis. This pointed towards the protective role of MC4R agonists in testicular ischemia, mediated via a cholinergic inflammatory pathway.

### 8.12. Role of Melanocortin Receptors in Insulin Resistance, Obesity, and Diabetes

In patients predisposed to develop type 2 diabetes mellitus, a brutal and endless cycle of obesity, insulin resistance, and diabetes exists. Melanocortin prevents the development of diabetes mellitus by affecting the central and peripheral metabolism and thus decreasing obesity and insulin resistance [168]. Leptin stimulates the POMC neuron, causing release of α-MSH, which acts on MC4R, increasing satiety and reducing obesity. Even in type 2 diabetes mellitus patients associated with leptin insensitivity, melanocortin evades the leptin pathway, increases satiety, and prevents the development of type 2 diabetes mellitus. α-MSH plays a dual role in this cycle. The presence of α-MSH in CNS enhances insulin sensitivity, whereas α-MSH causes insulin resistance in peripheral tissue [169]. In rat model, α-MSH diminishes abdominal fat and improves the action of insulin [170]. Melanocortin also has a peripheral effect. In the periphery, melanocortin acts via MC4R, activates brown adipose tissue, and increases lipolysis. Melanocortin also prevents lipogenesis caused by ASIP.

### 8.13. Role of Melanocortin Receptors in Pancreatic Diseases

**Figure 2:**
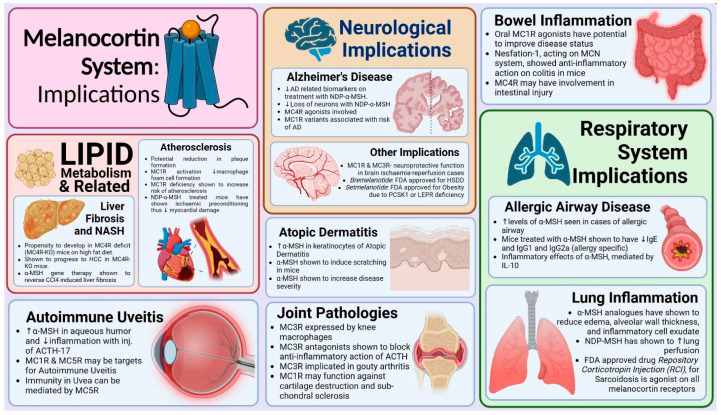
Summary of key implications of melanocortin system in various pathologies. ‘MC1R’—melanocortin receptor 1, ‘MC3R’—melanocortin receptor 3, ‘MC4R-KO’—melanocortin receptor 4 knock out, ‘MC5R’—melanocortin receptor 5, ‘NDP-α-MSH’—Nle4-D-Phe7-α-Melanocyte stimulating hormone, ‘HCC’— hepatocellular carcinoma, ‘CCl4’—carbon tetrachloride, ‘ACTH’—adrenocorticotropic hormone, ‘MCN’—melanocortin, ‘IL-10’—interleukin 10, ‘FDA’—Food and Drug Administration U.S.A.

Melanocortin receptors, specifically MC4R, play a role in pancreatitis. Wan et al. reported that melanocortin acts via MC3R and melanocortin antagonists diminishes the severity of cerulein-induced pancreatitis in rat models [171]. Jahovic et al. demonstrated that α-MSH protects the rats against acute pancreatitis [172]. A summary of all the key possible implications of the melanocortin system in various disorders has been given in Figure 2.

## 9. USFDA Approved New Drugs

The USFDA has approved several melanocortin agonists or antagonists in the management of different diseases. Table 2 demonstrates the current and prospective treatment of exogenous and endogenous melanocortin receptor antagonists and agonists to combat inflammatory phenotypes.

## 10. Conclusions

The melanocortin system is home to a fine balance of agonists, antagonists, and receptors. The complex interactions between agonistic peptides and the G protein-coupled receptors help in bringing about functions ranging from skin pigmentation (MC1R), steroid synthesis (MC2R), and energy balance (MC3R, MC4R) to immunomodulation (MC1R, MC5R). They also participate in both innate and acquired immune pathways. Genetic polymorphism in MC1R, MC2R, MC3R, and MC4R genes is associated with risk of melanoma, familial glucocorticoid deficiency, obesity, and type 2 diabetes mellitus, respectively. Collaborative involvement of the MC1R, MC2R, and MC5R genes are implicated in the risk of major depressive disorder. The immunomodulatory role of melanocortin and its receptors is well-known, but recently it has emerged as a potential anti-inflammatory agent [173]. Melanocortin receptors are involved in different inflammatory disorders, i.e., atopic dermatitis, autoimmune uveitis, sarcoidosis, respiratory diseases, multiple sclerosis, scleroderma, inflammatory bowel disease, amyotrophic lateral sclerosis, Alzheimer’s disease, arthritis, and reperfusion injury. Pharmacological agents revolving around melanocortin receptor-targeted ligands offer several advantages over the current anti-inflammatory drugs. They do not affect the host defense mechanism, for example, normal host microbiota, but rather enhance the immunological process and phagocytic activity and engage in various endogenous mechanisms to resolve inflammation. Overall, melanocortin receptor-based ligands engage endogenous pathways involved in the resolution of inflammation, thereby modulating rather than broadly suppressing immune responses. This mechanism may account for their potentially favorable safety profile compared with conventional immunosuppressants [174]. Several melanocortin agonists have recently received USFDA approval for specific indications, underscoring the translational potential of this pathway. Nevertheless, these therapies do not fully replicate the complexity of natural signaling, and their long-term safety and efficacy in inflammatory diseases remain to be established. With further refinement and clinical validation, melanocortin-based agents hold promise as future anti-inflammatory therapeutics.

## Figures and Tables

**Table 1:** Characteristic features of different melanocortin receptors and summary of melanocortin receptor polymorphisms and associated diseases.

Melanocortin Receptors	Key Polymorphisms	Tissue Distribution	Agonist	Antagonist	Function	Mutation
MC1R	rs1805007 (R151C), rs1805008 (R160W), rs1805009 (D294H), rs2228479 (V92M)	Melanocyte, keratinocyte, neutrophil, macrophage, monocyte	α-MSH, β-MSH, γ-MSH	ASIP	Melanin production and pigmentation, response to UV radiation, inflammation and immunomodulation	Increased risk of melanoma; Major depressive disorder (MDD)
MC2R	Rare mutations: S74I, D103N, R146H, nonsense variants	Adrenal cortex	ACTH	ACTH(7-38)	Adrenal gland function, production of cortisol	Familial glucocorticoid deficiency; Major depressive disorder (MDD)
MC3R	V81I, I172V	Hypothalamus and limbic system	α-MSH, β-MSH, γ-MSH	AGRP	Energy homeostasis, regulation of food intake and appetite, cell proliferation, neuronal regeneration, myocardial reperfusion	Risk of obesity
MC4R	Multiple loss-of-function variants	Brain, adipose tissue	α-MSH, β-MSH, γ-MSH	AGRP	Regulation of food intake and appetite, energy expenditure, thermogenesis	Most common monogenic cause of severe early-onset obesity type 2 diabetes
MC5R	Functional variants under investigation	Adrenal gland, adipose tissue, lung, liver	α-MSH, β-MSH, γ-MSH, ACTH		Stress response, cognitive function, and fetal brain development	Major depressive disorder (MDD)

**Table 2:** FDA-approved and investigational melanocortin receptor agonists/antagonists in inflammatory and metabolic disorders.

Generic Name (Brand)	Mechanism of Action	Primary Target(s)	Indication	Year of FDA Approval/Trial Status	Route of Administration	Trial Phase (If Investigational)	Known Adverse Effects
Bremelanotide (Vyleesi)	Nonselective melanocortin receptor agonist (MC4R, MC1R)	MC4R > MC1R	Hypoactive sexual desire disorder (HSDD)	Approved, 2019	Subcutaneous injection (on-demand, auto-injector)	–	Nausea, flushing, injection site reactions, headache, transient ↑ BP
Afamelanotide (Scenesse)	MC1R agonist; increases eumelanin production in skin	MC1R	Erythropoietic protoporphyria (EPP)	Approved, 2019	Subcutaneous implant (biodegradable, every 2 months)	–	Nausea, headache, hyperpigmentation, implant-site reactions
Setmelanotide (Imcivree)	Highly selective MC4R agonist (20× less activity at MC3R/MC1R)	MC4R	Chronic weight management in patients ≥ 6 years with POMC, PCSK1, or LEPR deficiency	Approved, 2020	Subcutaneous injection (daily)	–	Nausea, diarrhea, injection site reactions, depression, sexual adverse effects (erections)
Dersimelagon (MT-7117)	Oral selective MC1R agonist; increases eumelanin production	MC1R	Erythropoietic protoporphyria (EPP)	Investigational	Oral (capsule)	Phase III (EPP)	Nausea, hyperpigmentation, fatigue (reported in early-phase studies)
PL8177	Potent, selective MC1R agonist with anti-inflammatory properties	MC1R	Ulcerative colitis, inflammatory bowel disease	Investigational	Oral (enteric-coated)/subcutaneous	Phase II (UC)	GI symptoms, injection site pain (early studies)
AP1189 (Resomelagon)	Oral small-molecule biased MC1R/MC3R agonist	MC1R, MC3R	Rheumatoid arthritis, nephrotic syndrome	Investigational	Oral	Phase II (RA, nephrotic syndrome)	Mild GI symptoms, headache (early trials)

## Abbreviations

MCR	Melanocortin receptor
MSH	Melanocyte stimulating hormone
ACTH	Adrenocorticotropic hormone
POMC	Pro-opiomelanocortin
PC	Prohormone convertase
AGRP	Agouti-related protein
ASIP	Agouti-signaling protein
NF-kB	Nuclear factor kappa B
MHC	Major histocompatibility complex
CLIP	Corticotropin-like intermediate lobe peptide
LPH	Lipotropin
END	Endorphin
MRAP	Melanocortin receptor accessory protein
GPCR	G protein-coupled receptor
PKA	Protein kinase A
PKC	Protein kinase C
CAMP	Cyclic adenosine monophosphate
ERK	Extracellular signal-regulated kinase
MAPK	Mitogen activated protein kinase
PI3	Phosphoinositide 3-kinase
PLC	Phospholipase C
IP3	Inositol trisphosphate
CREB	Cyclic AMP response element binding protein
JAK/STAT	Janus kinase (JAK), signal transducer and activator of transcription protein (STAT)
PDZ domain	Postsynaptic density protein (PSD95), Drosophila disc large tumor suppressor (DlgA), and zonula occludens-1 protein (zo-1) domain
CCP	Clathrin-coated-pit
A2Ar	Adenosine 2 A receptor
ICAM	Intercellular adhesion molecule
SIRS	Systemic inflammatory response syndrome
USFDA	U.S. Food and Drug Administration
RCI	Repository corticotropin injection
NASH	Non-alcoholic steatohepatitis
